# Post-intensive care syndrome (PICS): recent updates

**DOI:** 10.1186/s40560-023-00670-7

**Published:** 2023-05-23

**Authors:** Stephanie L. Hiser, Arooj Fatima, Mazin Ali, Dale M. Needham

**Affiliations:** 1 Department of Health, Human Function, and Rehabilitation Sciences, The George Washington University, 2000 Pennsylvania Ave. NW, Suite 2000, Washington, DC 20006 USA; 2 Department of Physical Medicine and Rehabilitation, Johns Hopkins University School of Medicine, Baltimore, MD USA; 3 Outcomes After Critical Illness and Surgery (OACIS) Group, Johns Hopkins University, Baltimore, MD USA; 4 Division of Pulmonary and Critical Care Medicine, Department of Medicine, Johns Hopkins University School of Medicine, Baltimore, MD USA; 5 School of Nursing, Johns Hopkins University, Baltimore, MD USA

**Keywords:** Critical illness, Intensive care, Long-term outcomes

## Abstract

An increasing number of patients are surviving critical illness, but some experience new or worsening long-lasting impairments in physical, cognitive and/or mental health, commonly known as post-intensive care syndrome (PICS). The need to better understand and improve PICS has resulted in a growing body of literature exploring its various facets. This narrative review will focus on recent studies evaluating various aspects of PICS, including co-occurrence of specific impairments, subtypes/phenotypes, risk factors/mechanisms, and interventions. In addition, we highlight new aspects of PICS, including long-term fatigue, pain, and unemployment.

## Introduction

Intensive care units (ICUs) were established in the mid-1900s [1, 2]. With advances in life-saving interventions, survival improved over the past decades, positively impacting a large number of patients [1, 3, 4]. However, ICU survivors often report long-lasting impairments in physical, cognitive and/or mental health after hospital discharge [4]. In 2010, the Society of Critical Care Medicine (SCCM) convened an international multi-stakeholder group that created the term “Post-Intensive Care Syndrome” (PICS). PICS was created with multiple objectives, including: (1) to raise awareness among clinicians, patients/families and the general public, (2) to increase screening for specific impairments occurring after critical illness, (3) to facilitate further research into specific morbidities [4]. More specifically, PICS was defined “as new onset or worsening of impairment(s) in physical, cognitive, and/or mental health that arose after the ICU and persisted beyond hospital discharge” [4]. Furthermore, the PICS term can be applied to experiences of a family member (PICS-F) of a survivor of critical illness [4]. It is important to note that PICS is not a medical diagnosis, but a concept for improving education and awareness of post-ICU impairments [4].

Some recent publications highlighted in this narrative review evaluated data from the ARDSNet Long Term Outcomes Study (ALTOS). ALTOS is a multi-center study (including 41 hospitals in the USA) that prospectively examined physical, cognitive and mental health status at 6 and 12 months after Acute Respiratory Distress Syndrome (ARDS). This large study has recently expanded our understanding of PICS with data evaluating ICU survivors’ fatigue, pain, and delayed return to work [5]. In addition to highlighting these new data, this review is to present findings from additional recent PICS-related studies that focus on co-occurrence of specific morbidities, subtypes/phenotypes, risk factors, and interventions.

## General updates

### Incidence of post-ICU impairments

Determining the incidence of new or worsening impairments after critical illness is challenging due to a lack of data on pre-ICU baseline status [6]. As a result, most studies evaluate the prevalence of post-ICU impairments. However, a recent study of 2,345 ICU survivors in the Netherlands, collected baseline health status via questionnaires completed by patients or their proxies [6]. Among patients urgently admitted to the ICU, patients/proxies rated baseline health status retrospectively, while for those admitted for elective surgery, baseline questionnaires were disseminated at patients’ pre-operative visit and completed a few days before ICU admission [6]. Among those admitted to the ICU for medical (N = 649, 28%), urgent surgery (284, 12%), and elective surgery (1412, 60%), 58%, 64%, 43%, respectively, experienced new physical, cognitive and/or mental problems (Table 1) [6]. Notably, physical problems were measured using a non-validated questionnaire. The incidence of frailty, fatigue, muscle weakness, anxiety, depression, and cognitive impairment at 1 year post-ICU was more common among urgent surgical patients compared to elective surgery [6]. Patients undergoing elective surgery tended to have a shorter ICU length of stay than urgent surgery or medical patients [6]. Additionally, elective surgery patients were more likely to demonstrate improvements in physical and mental functioning at 1 year follow-up; however, baseline fatigue and anxiety were more common in elective surgery patients [6]. Overall, this landmark study provided new insights regarding the incidence of new impairments.

**Table 1:** Percentage of patients with new impairments at 1 year, by reason for admission

New Impairment	Reason for ICU admission		
	Urgent Surgery (n = 284)	Medical (n = 649)	Elective Surgery (n = 1,412)
Fatigue^a^	45	36	24
Depression symptoms^b^	20	18	10
Anxiety symptoms^b^	20	14	9
Frailty^c^	12	12	4
PTSD symptoms^d^	6	6	4

Data from a prospective cohort study across 4 hospitals in the Netherlands between 2016 and 2019 [7]

PTSD: post-traumatic stress disorder

^a^Measured using 8-item subscale of the 20-Item Checklist Individual Strength (CIS-20)

^b^Measured using the Hospital Anxiety and Depression Scale (HADS)

^c^Measured using the Clinical Frailty Scale (CFS)

^d^Measured using the Impact of Event Scale-Revised (IES-R)

An earlier smaller-sized study (N = 293) conducted in the United Kingdom (UK) found that ICU survivors experience more mobility issues, self-care issues, pain, and anxiety/depression after the ICU compared to their pre-ICU status based on the EQ-5D subscales [7]. However, this study is limited by potential for recall bias regarding baseline status and by use of only simple one-item assessments in the five subscales within the EQ-5D. Another earlier study (N = 36) conducted in the UK evaluated anxiety and depression symptoms among ICU survivors, excluding patients with pre-existing psychological symptoms; thus attempting to identify new symptoms after critical illness [8]. At 1 month after discharge, they found 16 (44%) and 17 (47%) of participants fell into the “disorder likely” category for anxiety and depression, respectively, based on scores from the Hospital Anxiety and Depression Scale (HADS) [8].

To better understand what long-term impairments are attributed to patients’ critical illness, we need further validation of methods of estimating baseline status [9, 10]. Additionally, future research should focus on evaluating the severity of impairments using continuous measures and via using validated and recommended measurement instruments [11], which would help have greater comparability in research findings and assist in understanding the magnitude of worsening of pre-existing impairments.

### Subtypes of physical, cognitive and mental health outcomes

To better understand PICS, researchers have conducted analyses to identify subtypes. From the ALTOS study with 698 ARDS survivors evaluated at 6- and 12-month follow-up, four subtypes were identified via weighted network analysis and recursive partitioning [12]: (1) mildly impaired physical and mental health status (22%), (2) moderately impaired physical and mental health status (39%), (3) severely impaired physical and moderately impaired mental health status (15%), and (4) severely impaired physical and mental health status (24%) [12]. As illustrated by these subtypes, physical and mental impairment, and severity of impairment, demonstrated close associations that were distinct from the presence and severity of cognitive impairment [12]. ICU-related variables and severity of illness were not associated with these subtypes of patient outcomes [12]. Notably, when considering retrospectively-assessed baseline status, patients in all four subtypes demonstrated declines from their baseline status.

Another recent study evaluating clustering of impairments among COVID-19 survivors reported that physical and mental impairments were closely related, but did not co-occur with cognitive impairments [13]. Notably, this study included both ICU and non-ICU patients. Another COVID-19 study evaluating outcomes at 1-year follow-up of ICU survivors reported that cognitive and mental impairments always occurred together [14].

Given common co-occurrence of physical and mental health impairments, future interventions should consider jointly targeting these impairments, such as considered with a novel behavioral activation-rehabilitation (the BEHAB trial) being evaluated via a pilot randomized trial [15]. Furthermore, distinct interventions targeting cognitive impairments are needed.

### Risk factors: patient/ICU specific

A multitude of risk factors for PICS-related impairments have been identified along with possible mechanisms for these impairments. A systematic review of 89 publications identified 60 risk factors, with approximately half categorized as patient-related and half as ICU-related [16]. Advanced age, female sex, a history of mental illness, severity of illness, poor ICU patient experience (including negative memories of the ICU), and delirium were significantly associated with physical, mental and/or cognitive impairments [16]. More specifically, a negative ICU patient experience and delirium have a strong impact on anxiety, Post-Traumatic Stress Disorder (PTSD), and cognitive function [16]. Although patient-related variables cannot be altered, they are helpful in identifying patients at highest risk for aspects of PICS. Interventions should target modifiable ICU-related risk factors; for instance, a negative ICU patient experience may be modified by implementing strategies to reduce delirium, increase early mobilization, optimize pain management, and reduce and/or modify the use of restraints [16]. The implementation of these strategies may facilitate alignment with patient-centeredness and improve patients’ ICU experiences; thus, addressing relevant risk factor for post-ICU impairments [17].

### Potential mechanisms: inflammatory subphenotypes

Recent research, using data from the ALTOS study, has explored the relationship between ICU-based hyper- vs. hypo-inflammatory subphenotypes with physical, cognitive and mental health impairments over 12-month follow-up [18]. The hyper-inflammatory phenotype was associated with decreased survival within 90 days [18]. However, survival did not differ beyond 90 days based on inflammatory phenotype [18]. Additionally, physical, cognitive, and mental outcomes at 6- and 12-month follow-up were similar across the two inflammatory subphenotypes [18].

Recent research also has demonstrated that acute systemic inflammation and coagulation markers measured early in critical illness are not associated with cognitive function at 3 and 12-month follow-up. Moreover, only 2 markers were associated with disability in activities of daily living over follow-up [19].

Hence, based on these two studies, inflammation during critical illness may not an appropriate mechanistic target for future intervention. However, evaluating associations of prolonged inflammation after hospital discharge with PICS-related impairments merits more investigation [20].

### Interventions

A recent systematic review of 36 studies with 5,165 patients, evaluated the effectiveness of non-pharmacological interventions for improving long-term outcomes after critical illness [21]. The study classified interventions into early mobilization and physical rehabilitation (56%), post-ICU follow-up (14%), psychosocial programs (8%), ICU diaries (8%), and educational activities (6%) [21]. Results from each of these 36 studies are summarized in Table 2 [8, 21–56]. Only 31% of these studies included interventions after hospital discharge. Given the prolonged impairments experienced by patients, further studies evaluating the impact of interventions post-discharge are needed [21]. Notably, existing studies have risk of bias from incomplete reporting and loss to follow-up, along with lack of standardization in instruments used to measure outcomes [21]. Hence, further improvement in study design is needed. Overall, the design and evaluation of non-pharmacological interventions targeting aspects of PICS is at an early stage and needs further investigation to improve our understanding of potential efficacy.

**Table 2:** Evaluations of non-pharmacological interventions for improving long-term outcomes after critical illness

Study (County, Year, Sample Size^a^)	Summary of results at last follow-up time point (intervention group vs. control group)
Pre-Hospital Exercise	
Arthur et al. [32] (Canada, 2000, N = 249)	Hospital LOS [Median (IQR)]: 6 (5–7) vs. 7 (6–8) days; MD (95% CI) 1.0 (0.0 to 1.0), p = 0.002 Mean (SD) change from baseline SF-36 Physical Component Summary Score during the pre-operative phase: 1.6 (7.5) vs. − 1.5 (7.8); MD (95% CI) 3.0 (1.0 to 5.0)
In-ICU Exercise/Physical Rehabilitation	
Wright et al. [43] (UK, 2018, N = 308)	Mean (SD) SF-36 Physical Component Summary Score at 6 months: 37 (12) vs. 37 (11). MD (95% CI) − 1.1 (− 7.1 to 5.0)
Morris et al. [51] (USA, 2016, N = 300)	Mean (95% CI) SF-36 Physical Functioning scale score at 6 months: 56 (50–62) vs. 44 (38–50). MD (95% CI) 12 (3.8 to 21), p = 0.001 Mean (95% CI) Functional Performance Inventory score at 6 months: 2.2 (2.1–2.4) vs. 2.0 (1.9–2.2). MD (95% CI) 0.2 (0.04 to 0.4), p = 0.02 Mean (95% CI) Short Physical Performance Battery at 6 months: 9.0 (8.3–9.7) vs. 8.0 (7.2–8.7). MD (95% CI) 1.1 (0.04 to 2.1), p = 0.04
Hodgson et al. [52] (Australia/New Zealand, 2016, N = 50)	Mean (SD) EQ-5D score at 6 months: 0.63 (0.27) vs. 0.63 (0.33), p = 0.25 Mean (SD) IADL score at 6 months: 7 (2) vs. 7 (1), p = 0.81 Mean (SD) HADS score at 6 months: 12 (9) vs. 11 (7), p = 0.91
Schaller et al. [53] (Austria/Germany/USA, 2016, N = 200)	Median (IQR) mini-modified FIM at hospital discharge: 8 (4–8) vs. 5 (2–8). MD (95% CI) 3.0 (1.0 to 4.0), p = 0.0002
Kayambu et al. [54] (Australia, 2015, N = 50)	Mean (SD) SF-36 Physical function score at 6 months: 82 (22) vs. 60 (29), p = 0.04 Mean (SD) SF-36 Physical role score at 6 months: 61 (44) vs. 17 (34), p = 0.005
Moss et al. [55] (USA, 2016, N = 120)	Mean (SD) Continuous Scale Physical Functional Performance Test short form (CS-PFP-10) at 6 months: 40 (4) vs. 44 (4), p = 0.43
Denehy et al. [56] (Australia, 2013, N = 150)	Mean (SE) 6MWT (meters) at 12 months: 410 (23) vs. 405 (23). MD (95% CI) 4.7 (− 60 to 69), p = 0.88 Mean (SD) TUG (seconds) at 12 months: 10 (6.2) vs. 14 (25). MD (95% CI) − 7.3 (− 19 to 4.4), p = 0.22) Mean (SD) Assessment of Quality of Life Measure at 12 months: 0.5 (0.4) vs. 0.5 (0.4). MD (95% CI) 0.0 (− 0.1 to 0.2), p = 0.75 Mean (SD) SF-36 Physical function score at 12 months: 41 (13) vs. 44 (11). MD (95% CI) 1.6 (− 3.7 to 7), p = 0.54 Mean (SD) SF-36 Physical Component Summary score at 12 months: 45 (11) vs. 46 (9). MD (95% CI) 0.3 (− 4.3 to 4.8), p = 0.9 Mean (SD) SF-36 Mental Component Summary score at 12 months: 48 (12) vs. 45 (16). MD (95% CI) 5 (− 1.1 to 11.1), p = 0.12
Chen et al. [22] (Taiwan, 2010, N = 34)	Median (IQR) Total FIM score at 1 year: 78 (62–126) vs. 31 (21–50), p < 0.001
Post-ICU Exercise/Physical Rehabilitation	
Vitacca et al. [23] (Italy, 2016, N = 48)	Mean (95% CI) change in maximal inspiratory pressure (cmH_2_O) at 6 months: 14 (5.8–22) vs. − 0.2 (− 7.8 to 7.4), p = 0.007 Mean (95% CI) change in Basic Activities of Daily Living at 6 months: 1 (0–4) vs. 1 (0–4), p = 0.63 Mean (95% CI) change in EQ-5D score at 6 months: 0.23 (− 0.29 to 0.73) vs. 0.032 (− 0.29 to 0.24), p = 0.04
Brummel et al. [24] (USA, 2013, N = 87)	*3 Groups: Cognitive + *Physical Therapy vs. Physical Therapy vs. Usual Care* Median (IQR) Tower test (executive functioning) at 3 months: 10 (8–11) vs. 11 (11–12) vs. 10 (9–12), p = 0.2 Median (IQR) Dysexecutive questionnaire (executive functioning) at 3 months: 9 (2–18) vs. 10 (5–17) vs. 18 (9–29), p = 0.08 Median (IQR) Mini-mental state exam (global cognition) at 3 months: 29 (28–30) vs. 29 (27–30) vs. 28 (27–29), p = 0.64 Median (IQR) TUG (functional mobility) at 3 months: 11 (9–13) vs. 10 (8–13) vs. 8 (8–14), p = 0.79 Median (IQR) Katz ADL at 3 months: 0 (0–2) vs. 0 (0–1) vs. 0 (0–0), p = 0.69 Median (IQR) EQ-5D at 3 months: 75 (54–80) vs. 80 (62–89) vs. 75 (61–86), p = 0.44
Jones et al. [25] (UK, 2003, N = 126)	SF-36 Physical function scores at 6 months. when controlling for length of ICU stay, were significantly different between group, p = 0.006 (numerical scores not reported) Number (%) of patients HAD anxiety scale score > 11 at 6 months: 19 (33%) vs. 15 (34%), p = 0.71
Battle et al. [26] (UK, 2019, N = 60)	Mean (SE) 6MWT at 12 months: 345 (63) vs. 295 (57). MD (95% CI) -50 (-224 to 124), p = 0.37 Mean (SE) HAD-A at 12 months: 4 (1) vs. 9 (1). MD (95% CI) − 4 (1 to 5), p = 0.006 Mean (SE) HAD-D at 12 months: 5 (1) vs. 7 (1). MD (95% CI) − 3 (1 to 3), p = 0.11
Shelly et al. [27] (India, 2017, N = 35)	Median (IQR) difference in SF-36 Physical component summary at 4 weeks: 10.3 (8.5–14.9) vs. 7.4 (3.7–8.5), p = 0.003 Median (IQR) difference in SF-36 Mental component summary at 4 weeks: 21.8 (15.7–24.1) vs. 14.1 (10.8–19.5), p = 0.006
McDowell et al. [28] (UK, 2016, N = 60)	Mean (SD) change in SF-36 Role physical score at 6 weeks: 12 (9.8) vs. 5.4 (12). MD (95% CI) 6.6 (0.73 to 12.5), p = 0.03 Mean (SD) change in Incremental Shuttle Walk Test (meters) at 6 weeks: 136 (120) vs. 52 (127). MD (95% CI) 83 (8.3 to 158), p = 0.03 Mean (SD) change in functional limitation profile score at 6 weeks: − 7.8 (7.4) vs. − 3.0 (6.3). MD (95% CI) − 4.8 (− 8.7 to − 0.9), p = 0.02
McWilliams et al. [29] (UK, 2015, N = 63)	Mean (95% CI) change in SF-36 Physical component summary score at 8–10 weeks: 8.6 (5.4 to 10.6) vs. 3.5 (1.6 to 6.7) Mean (95% CI) change in SF-36 mental component summary score at 8–10 weeks: 10 (6.9 to 14) vs. 4.3 (0.5 to 7.6)
Connolly et al. [30] (UK, 2015, N = 20)	Median (IQR) change in Incremental Shuttle Walk Test (meters) at 3 months: 115 (− 3 to 238) vs. 170 (40 to 315) Median (IQR) change in 6MWT (meters) at 3 months: 140 (36 to 210) vs. 185 (40 to 285) Median (IQR) change in SF-36 Physical component summary at 3 months: 2 (− 7 to 16) vs. 11 (4 to 28) Median (IQR) change in SF-36 Mental component summary at 3 months: 14 (− 3 to 27) vs. 11 (− 19 to 19) Median (IQR) change in HADS at 3 months: − 6 (− 9 to 3) vs. − 5 (− 13 to − 3)
Batterham et al. [31] (UK, 2014, N = 59)	Mean (SD) anaerobic threshold at week 26: 13 (18) vs. 12 (20). MD (95% CI) of 0.6 (− 1.6–2.8) Mean (SD) SF-36 Physical function score at week 26: 47 (21) vs. 47 (25). MD (95% CI) of 0.1 (− 6.0 6.2) Mean (SD) SF-36 Mental health score at week 26: 51 (21) vs. 47 (25). MD (95% CI) 4.4 (− 2.4 to 11.2)
Jackson et al. [33] (USA, 2012, N = 21	Median (IQR) Tower test at 3 months: 13 (12–14) vs. 7.5 (4.0–8.5), adjusted p < 0.01 Median (IQR) TUG at 3 months: 9 (9–12) vs. 10 (9–12), adjusted p = 0.51 Median (IQR) Functional Activities Questionnaire Score at 3 months: 1.0 (0.0–3.0) vs. 8.0 (6.0–12), adjusted p = 0.04 Median (IQR) Dysexecutive questionnaire (executive functioning) at 3 months: 8 (6–14) vs. 16 (8–19), adjusted p = 0.74 Median (IQR) Mini-mental state exam (global cognition) at 3 months: 30 (29–30) vs. 27 (25–29), adjusted p = 0.25
Elliott et al. [34] (Australia, 2011, N = 195)	Mean (95% CI) SF-36 Physical function score at 26 weeks: 15 (12–18) vs. 14 (11–16). MD (95% CI) 1 (− 3 to 5), effect size 0.08 Mean (95% CI) 6MWT at 26 weeks: 126 (99–153) vs. 116 (86–147). MD (95% CI) 9.6 (− 31 to 51), effect size 0.08 Mean (95% CI) SF-36 Physical component summary score at 26 weeks: 11 (8–14) vs. 11 (8–13). MD (95% CI) 0 (− 3 to 4), effect size 0.03 Mean (95% CI) SF-36 Mental component summary score at 26 weeks: 10 (6–13) vs. 8 (5–11). MD (95% CI) 2 (− 3 to 6), effect size 0.10
Follow-up services	
Jonasdottir et al. [35] (Iceland, 2018, N = 168)	Mean (SD) HADS-A total score at 12 months: 4 (3) vs. 2.5 (2.8), p = 0.005 Mean (SD) HADS-D total score at 12 months: 4 (3) vs. 4 (4), p = 0.895 Mean (SD) IES-R Score at 12 months: 20 (17) vs. 14 (15), p = 0.066
Jensen et al. [36] (Denmark, 2016, N = 386)	MD (95% CI) SF-36 Physical component summary score at 12 months: 1.4 (− 1.5 to 4.4), p = 0.35 MD (95% CI) SF-36 Mental component summary score at 12 months: 1.9 (− 1.1 to 4.9), p = 0.21 MD (95% CI) HADS-A at 12 months: − 0.21 (− 1.22 to 0.80), p = 0.68 MD (95% CI) HADS-D at 12 months: − 0.20 (− 1.12 to 0.72), p = 0.67 MD (95% CI) HTQ-IV score (PTSD severity) at 12 months: − 1.42 (− 3.94 to 1.11), p = 0.27
Schmidt et al. [37] (Germany, 2016, N = 143)	MD (SD) difference in SF-36 Physical component summary score at 12 months: 10 (12) vs. 8 (14). MD (95% CI) 1.1 (− 2.7 to 4.9), p = 0.56 MD (SD) difference in SF-36 Mental component summary score at 12 months: 4 (13) vs. 2 (13). MD (95% CI) 1.4 (− 2.4 to 5.2), p = 0.47
Cuthbertso et al. [38] (UK, 2009, N = 286)	Mean (SD) SF-36 Physical component summary score at 12 months: 42 (11) vs. 41 (12). MD (95% CI) 1.1 (− 1.9 to 4.2), p = 0.46 Mean (SD) SF-36 Mental component summary score at 12 months: 47 (13) vs. 47 (12). MD (95% CI) 0.4 (− 3.0 to 3.7), p = 0.83 Mean (SD) EQ-5D Utility score at 12 months: 0.58 (0.37) vs. 0.60 (0.30). MD (95% CI) 0 (− 0.1 to 0.1), p = 0.57 Mean (SD) HADS-A score at 12 months: 6 (5) vs. 6 (4). MD (95% CI) − 0.8 (− 1.9 to 0.4), p = 0.57
Douglas et al. [39] (USA, 2007, N = 335)	No difference in SF-8 physical scores at 2 months, p = 0.40, controlling for baseline scores and APACHE III No difference in SF-8 mental scores at 2 months, p = 0.22, controlling for baseline scores and APACHE III
Psychosocial Programs	
Cox et al. [40] (USA, 2018, N = 80)	Mean (95% CI) change from baseline for PHQ-9 at 3 months: Telephone group (TG) − 3.9 (− 5.6 to − 2.2), Mobile group (MG) − 4.8 (− 6.6 to − 2.9), Education group (EG) − 3.0 (− 5.3 to 0.8). MD (95% CI) TG vs. EG − 0.9 (− 3.7 to 2.0), p = 0.41. MD (95% CI) MG vs. EG − 1.7 (− 4.7 to 1.2), p = 0.25 Mean (95% CI) change from baseline for GAD-7 at 3 months: TG − 1.6 (− 3.0 to − 0.1), MG − 2.1 (− 3.7 to − 0.5), EG − 0.6 (− 2.5 to 1.3). MD (95% CI) TG vs. EG − 1.0 (− 3.3 to 1.4), p = 0.43. MD (95% CI) MG vs. EG − 1.5 (− 3.9 to 1.0), p = 0.24 Mean (95% CI) change from baseline for PTSD at 3 months: TG − 2.2 (− 5.6 to 1.2), MG − 2.6 (− 6.3 to 1.2), EG − 3.5 (− 8.0 to 1.0). MD (95% CI) TG vs. EG 1.3 (− 4.4 to 7.0), p = 0.65. MD (95% CI) MG vs. EG − 0.9 (− 4.9 to 6.8), p = 0.75 Mean (95%) change from baseline for PHQ-15 at 3 months: TG − 3.7 (− 5.2 to − 2.2), MG − 5.3 (− 7.0 to − 3.7), EG − 4.8 (− 6.8 to 2.7). MD (95% CI) TG vs. EG 1.1 (− 1.5 to 3.6), p = 0.41. MD (95% CI) MG vs. EG − 0.6 (− 3.2 to 2.0), p = 0.52
Cox et al. [41] (USA, 2017, N = 175)	Mean (SE) HADS at 6 months: 16 (1) vs. 16 (1). MD (95% CI) − 0.3 (− 2.7 to 2.0), p = 0.78 Mean (SE) IES-R at 6 months: 29 (3) vs. 26 (3). MD (95% CI) 3.6 (− 2.7 to 10.0), p = 0.26 Mean (SE) EQ-5D at 6 months: 61 (3) vs. 61 (3). MD (95% CI) 0.3 (− 5.9 to 6.6), p = 0.92
Agren et al. [42] (Sweden, 2014, N = 84)	*No difference* in SF-36 between groups at 12 months (numeric data and p-value not reported)
Diaries	
Garrouste et al. [44] (France, 2012, N = 216)	Mean (SD) of IES-R data score at 12 months pre-diary 35 (16), diary 21 (12), post diary 30 (15), p = 0.03
Jones et al. [45] (6 European countries, 2010, N = 352)	Incidence of PTSD 5% vs 13%, p = 0.02
Knowles et al. [8] (UK, 2009, N = 36)	Number (%) of patients with HADS-anxiety score ≥ 8 at ~ 7 weeks: 2 (11) vs. 7 (39), p < 0.05 Number (%) of patients with HADS-depression score ≥ 8 at ~ 7 weeks: 3 (17) vs. 8 (44), p < 0.07
Information & Education Programs	
Demircelik et al. [46] (Turkey, 2016, N = 100)	Mean (SD) in change in HADS (anxiety) from ICU to 1 week discharge: 4.2 (0.58) vs. 0.6 (0.42), p < 0.01 Mean (SD) in change in HADS (depression) from ICU to 1 week discharge: 3.5 (0.53) vs. 0.3 (0.46), p < 0.01
Fleisher et al. [47] (Germany, 2014, N = 211)	Mean (SD) SF-12 Physical component summary score at 3 months: 41 (9) vs. 40 (10). MD (95% CI) 0.3 (− 3.1 to 3.6), p = 0.87 Mean (SD) SF-12 Mental component summary score at 3 months: 47 (11) vs. 48 (11). MD (95% CI) − 1.3 (− 5.3 to 2.6), p = 0.5
ABCDE Bundle	
Sosnowski et al. [48] (Australia, 2018, N = 30)	Mean (SD) SF-36 Physical component summary score at 90 days: 44 (12) vs. 38 (11) Mean (SD) SF-36 Mental component summary score at 90 days: 47 (16) vs. 40 (16)
Earplugs and eye mask during ICU	
Demoule et al. [49] (France, 2017, N = 64)	Median (IQR) HADS-A at 90 days: 8 (4–11) vs. 6 (4–12), p = 0.69 Median (IQR) HADS-D at 90 days: 6 (3–12) vs. 6 (2–9), p = 0.63 Median IES-R at 90 days: 11 (5–18) vs. 16 (9–27), p = 0.15
Structured mirrors during ICU	
Giraud et al. [50] (UK, 2016, N = 223)	Mean (SD) EQ-5D at 12 weeks: 73 (19) vs. 77 (15), p = 0.13

LOS: Length of stay; IQR: Interquartile range; MD: Mean Difference; SD: Standard deviation; EQ-5D: EuroQol-5 Dimension; IADL: Instrumental Activities of Daily Living; HADS: Hospital Anxiety and Depression Scale; FIM: Functional independence measure; 6MWT: 6 min-walk-test; TUG: Timed Up and Go; HRQOL: Health related quality of life; IES-R: Impact of Event Scale-revised; PHQ-9: Patient health questionnaire-9; GAD-7: General anxiety disorder-7; PHQ-15: Patient health questionnaire-15; PTSD: post-traumatic stress disorder; HTQ-IV: Harvard Trauma Questionnaire Part IV

Studies identified based on systematic review by Geense et al. [21]

^a^Sample size = total patients enrolled in the study

## Recent data on additional aspects of PICS

### Fatigue

Survivors of acute respiratory failure commonly experience fatigue with growing empirical evaluation of this symptom. An analysis of data from the ALTOS study (n = 732) evaluated fatigue symptoms using the validated Functional Assessment of Chronic Illness Therapy-Fatigue Scale (FACIT-F) [5], with 70% and 66% reporting fatigue at 6 and 12 months respectively [5]. At 12-month versus 6-month follow-up, 28% of participants reported their symptoms were worse, 31% reported no change, and 41% reported improved symptoms. Increased fatigue was associated with female sex and unemployment prior to hospital admission [5]. At 6 and 12 months, patients with fatigue symptoms had worse physical functioning and higher psychological impairments [5]. Thus, health care providers should screen for both physical and psychological impairments among ICU survivors reporting fatigue. Importantly, in this cohort of ARDS survivors, there was no association between fatigue and ICU length of stay or severity of illness [5]. Additionally, a prospective study among a broader population of ICU survivors, rather than exclusively ARDS survivors, reported a high prevalence of fatigue at 12 month follow-up among medical, urgent surgery, and elective surgery ICU survivors as follows: 36%, 45%, and 24%, respectively [6].

### Pain

In the ALTOS study, nearly 50% of ARDS survivors reported clinically significant pain during the first year after ARDS [57]. Unemployment and the use of opioids in the ICU were associated with greater pain at 6- and 12-month follow-up [57]. Among those with pain, 78% also reported anxiety and/or depressive symptoms and 78% reported cognitive and/or physical function impairment. This prevalence in the ALTOS study was similar to another study that reported 31% and 35% of medical and surgical ICU survivors having moderate to severe pain at 3 and 12 months, respectively [58]. In contrast, the prevalence of pain in the community is substantially lower, with only 20% of the US population reporting chronic pain [15]. A prior study using the brief pain inventory (BPI) measurement instrument in 295 patients from medical and surgical ICUs examined pain intensity and its effect on patients after hospital discharge [58]. Cumulative ICU opioid exposure was not associated with increased pain intensity or increased pain interference of daily life after the intensive care unit [58]. The authors suggest that patients with underlying chronic pain may report higher pain after hospital discharge due to opioid tolerance, hyperalgesia, or predisposition to developing a pain syndrome [58].

### Delayed return to work and joblessness

**Fig. 1:**
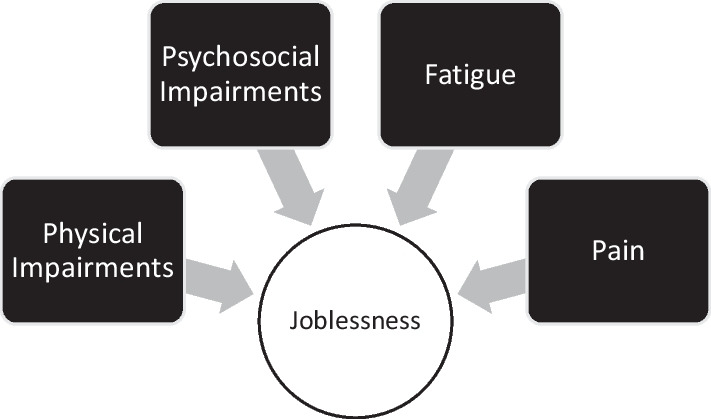
Barriers to return to work after critical illness

Previously-employed survivors of critical illness experience challenges in returning to work after hospital discharge (Fig. 1) [59]. Some issues commonly encountered are delayed return to work, loss of job after return to work, and the need to change occupations [59, 60]. These problems frequently lead to a financial burden for patients and their families [59]. A meta-analysis, including 52 studies evaluating 10,015 previously-employed ICU survivors, assessed return to work [60]. Approximately 36%, 64%, 60% of patients reported return to work at 1 to 3, 6, and 12- month follow-up, respectively [60]. Furthermore, results from the ALTOS study, including 326 previously-employed ARDS survivors, found that 48% and 43% were jobless at 6- and 12-month follow-up [61]. Patients with pain or fatigue were less likely to return to work [61]. At 6 and 12 months, the imbalance between occupational workload requirements and ARDS survivors’ functional ability occurred in 90% of ALTOS participants [62]. Furthermore, having imbalance in both physical and psychosocial areas at 6 months was significantly associated with joblessness at 6 and 12 months [62]. The findings from these studies highlight the need to improve patient’s functional abilities, and to decrease work load via workplace accommodations for ICU survivors [62, 63].

## Conclusion

Post-intensive care syndrome is experienced by many ICU survivors who have new or worsening physical, cognitive, and/or mental health impairments. These impairments often co-occur and may include pain and fatigue. Together these impairments and symptoms create substantial challenges in returning to work for previously-employed ICU survivors. Evaluation of ARDS survivor subtypes/phenotypes demonstrate that physical and mental health impairments are closely associated, without association with cognitive outcomes. The biological mechanisms underlying many of these long-standing impairments are uncertain despite exploration into inflammatory biomarkers in the ICU setting. Increased understanding of risk factors, especially across different types of ICU patients has improved our ability to potentially identify high-risk patients for screening and intervention. However, in terms of interventions, evaluation of non-pharmacological interventions, including early mobilization and physical rehabilitation in the ICU, ICU diaries, psychological interventions, multi-disciplinary post-ICU follow-up clinics and interventions, and educational activities, are still in an early stage. Future well-designed studies are needed to better understand mechanisms and potential interventions to improve post-intensive care syndrome.

## Data Availability

Not applicable.

## Abbreviations

ALTOS: ARDSNet Long Term Outcomes Study
ARDS: Acute Respiratory Distress Syndrome
BPI: Brief pain intensity
COVID-19: Coronavirus disease 2019
FACIT-F: Functional assessment of chronic illness therapy fatigue scale
HADS: Hospital Anxiety and Depression Scale
ICU: Intensive Care Unit
PICS: Post-intensive care syndrome
PTSD: Post-traumatic stress disorder
SCCM: Society of Critical Care Medicine
UK: United Kingdom
